# Validated nomograms for non-metastatic colorectal cancer prognosis prediction: a population-based study

**DOI:** 10.3389/fonc.2025.1691693

**Published:** 2025-10-24

**Authors:** Yu-Dan Cao, Hong-Wei Sun, Tian-Ci Li, Jia-Rui Xie, Xin-Ran Shi, Qian Chen, Yue Chen, Zi-Yang Peng, Quan-Peng Qiu, Nuo-Xuan Niu, Jun-Jun She, Xiang Li

**Affiliations:** ^1^ Department of Otorhinolaryngology Head and Neck Surgery, The First Affiliated Hospital of Xi’an Jiaotong University, Xi’an, Shaanxi, China; ^2^ Department of High Talent, Center for Gut Microbiome Research, Med-X Institute Centre, The First Affiliated Hospital of Xi’an Jiaotong University, Xi’an, Shaanxi, China; ^3^ Department of General Surgery, The First Affiliated Hospital of Xi’an Jiaotong University, Xi’an, Shaanxi, China

**Keywords:** Alb-dNLR score, nomogram, non-metastatic colorectal cancer, prognosis, inflammation, nutrition

## Abstract

**Introduction:**

The TNM staging system has limitations in predicting prognosis for colorectal cancer (CRC). This study aimed to develop and validate a nomogram incorporating the albumin-derived neutrophil-to-lymphocyte ratio (Alb-dNLR) score, a novel nutritional and inflammatory biomarker, to provide a more accurate and personalized prognostic prediction for patients with non-metastatic CRC.

**Methods:**

This retrospective study included a development cohort of 457 non-metastatic CRC patients who underwent radical colectomy, and an external validation cohort of 207 patients. Optimal cut-off values for continuous variables were determined by ROC curve analysis. Multivariable Cox and LASSO regression models were used to identify independent prognostic factors for cancer-specific survival (CSS) and to construct two nomograms. The performance of the nomograms was assessed by C-indexes, calibration plots, and decision curve analysis (DCA).

**Results:**

Multivariable analysis confirmed that a high Alb-dNLR score was an independent predictor for worse cancer-specific survival (CSS) (HR = 5.536, P < 0.001) and overall survival (OS) (HR = 4.690, P < 0.001). Both nomograms, developed from Cox and LASSO models, showed superior discrimination compared to the TNM staging system alone (C-index in development cohort: (0.785, 0.767 vs. 0.680). Calibration plots and DCA confirmed the nomograms’ accuracy and clinical utility in both cohorts.

**Conclusions:**

The Alb-dNLR score is a simple and effective independent prognostic biomarker for non-metastatic CRC. The nomograms incorporating Alb-dNLR score provide a more accurate and practical tool than the TNM system for predicting patient survival, thereby facilitating personalized clinical decision-making.

## 1 Introduction

Colorectal carcinoma (CRC) ranks as the fourth most deadly cancer in the world, accounting for 10% of all cancer diagnoses, with nearly 900,000 deaths each year (1, 2). In the absence of initial symptoms, a considerable proportion of CRC patients are diagnosed at the progressive stage, leading to unfavorable survival outcomes (3). Therefore, it is essential to predict the prognosis of CRC patients at an early stage.

Currently, the TNM staging system by American Joint Committee on Cancer (AJCC) (8th edition) is widely used for evaluating the development and prognosis of most malignancies, including CRC, and guiding treatment options. However, prognostic heterogeneity exists among patients in the same TNM stage (4). This may be because the TNM system only assesses pathological features but ignores basic information (e.g., sex, age) and physical status (e.g., nutritional and inflammatory status), potentially leading to therapeutic uncertainty. Therefore, it is of interest to integrate additional prognostic indicators and customized models into clinical practice to enhance prognostic prediction accuracy and to advance personalized therapeutic strategies.

Previous studies have shown that various factors can influence cancer development, including inflammation levels, nutritional status, and immune function. Inflammation promotes angiogenesis, tumor growth, invasion, and metastasis (5, 6). Accordingly, in recent years, a set of indices have been established to predict outcomes for CRC patients (7, 8), including neutrophil-to-lymphocyte ratio (NLR) (9), and derived NLR (dNLR) (10, 11). However, these biomarkers primarily consider the immunity-inflammation interactions while neglecting the potential influence of the patient’s general and nutritional status on prognosis. Moreover, individual biomarkers have limited discrimination and inaccuracy, as single-dimensional indices are more prone to external interference.

Albumin is a common marker in liver function tests and is routinely used to display patients’ nutritional status. The combination of albumin and inflammatory status has been demonstrated to correlate significantly with the prognosis of patients diagnosed with cancers. Some novel indices that combined nutritional and immunological status indexes have proved their prognostic value in various cancers, such as prognostic nutritional index (PNI) (12), C-reactive protein-albumin-lymphocyte (CALLY) (13), and modified Glasgow prognostic score (mGPS) (14). However, these nutritional-inflammatory indices are complicated to calculate and require data from multiple tests and are not suitable for clinical practice. To maximize convenience for clinical utility, we focused on albumin-derived neutrophil-to-lymphocyte ratio (Alb-dNLR) score (15), which only requires albumin level, neutrophil, and leukocyte count and has been investigated to be an independent prognostic factor for esophageal squamous cell cancer (ESCC) (16) and rectal cancer (17). However, few studies have been conducted to examine its predictive capacity in non-metastatic CRC patients.

To date, most of the parameter selection processes in studies are based on univariate and multivariate Cox regression analysis, which are at risk of causing multicollinearity-related bias between variables (18). To solve this problem, the least absolute shrinkage and selection operator (LASSO) algorithm based on machine learning has been applied in our study, as it can build a more refined model, and its accuracy has been confirmed in previous research (19, 20). In this study, we conducted comprehensive multivariate analyses utilizing Cox and LASSO regression models, respectively.

This study aimed to establish and validate the prognostic value of nomograms based on Alb-dNLR score in predicting the prognosis of non-metastatic CRC patients, depicting risk factors, and visually assisting in clinical decision-making.

## 2 Methods and materials

### 2.1 Patients

Our study screened 513 patients who underwent colectomy for CRC at our institution for eligibility from April 2013 to April 2019. The inclusion criteria were: (i) pathologically confirmed CRC diagnosis; (ii) no distant metastases occur; (iii) treatment included a radical surgery, either alone or with postoperative chemotherapy (CT) or chemoradiotherapy (RCT). Exclusion criteria were: (i) patients with synchronous malignancies; (ii) the presence of hematological or primary liver diseases; (iii) pre-treatment neoadjuvant therapy.

In the end, 457 cases were included in the development cohort. An additional validation cohort consisting of 207 patients was established in accordance with aforementioned criteria. Informed consent was signed by the patients themselves or their guardians.

### 2.2 dNLR and Alb-dNLR score

The dNLR was constructed as follows: neutrophil count/(leukocyte count-neutrophil count). Cut-off values for serum albumin level and dNLR were determined through receiver operating characteristics (ROC) analysis, based on which patients were categorized as follows: those with both low albumin levels and elevated dNLR are assigned a score of 2, those with either of the two abnormalities receive a score of 1, and patients with high albumin levels and low dNLR are given a score of 0.

### 2.3 Data collection

The Biobank of First Affiliated Hospital of Xi’an Jiaotong University was utilized for the extraction of patients’ demographic features (i.e., sex, age), life history (e.g. smoking status), and clinicopathological features (i.e., treatment strategy, tumor location, tumor differentiation, TNM stage). All patients were restaged according to the 8th edition of TNM staging system (21). Serum albumin levels, neutrophil counts, and leukocyte counts were obtained through hematologic assessments, including blood routine tests and liver function evaluations performed within one week prior to radical surgery.

### 2.4 Treatment

The treatment approach was customized following thorough deliberation by a multidisciplinary team in accordance with the National Comprehensive Cancer Network (NCCN) guidelines. Radical surgeries were performed on all patients in our study. The initial chemotherapy regimen comprised FOLFOX and CAPOX regimens, with 5-FU/LV or capecitabine monotherapy administered to patients unable to tolerate intravenous therapy.

### 2.5 Follow up

All patients were regularly followed up in the outpatient clinic until death or the final follow-up on April 1, 2024. Physical exams and laboratory tests, including CEA and CA19-9, were conducted every 3–6 months. Imaging tests such as chest X-rays, CT scans or ultrasounds of the abdomen and pelvis, and colonoscopies were conducted in accordance with the NCCN guidelines. The primary endpoint was cancer-specific survival (CSS), regarded as the time span from the date of initial diagnosis to death due to recurrence or final follow-up (on April 1, 2024), whichever came first. Overall survival (OS) is the secondary endpoint of interest, calculated as the duration (in months) from the initial diagnosis to either the date of death for all cases or until the final follow-up (on April 1, 2024).

### 2.6 Statistical analysis

All statistical analyses in the present study were conducted using R software (version 4.3.3; http://www.Rproject.org) and SPSS software (version 26.0). Receiver operating characteristics (ROC) curves were plotted, and the optimal cut-off values for serum albumin level and dNLR were calculated. Categorical variables were presented as frequencies and percentages, and their differences were calculated using the χ^2^ test. Kaplan–Meier curves and log-rank tests were employed to analyze the cumulative incidence of events. Univariate analyses were carried out with Cox proportional hazard model. Multivariable analyses utilizing Cox and LASSO models were performed to assess the independent prognostic value of each variable for CSS and OS. Nomograms predicting the 1-, 3- and 5-year CSS were constructed by incorporating independent factors in multivariable Cox and LASSO models. The concordance index (C-index) and areas under the time-ROC curves (AUC) were calculated to evaluate the performance for discrimination of nomograms. Calibration curves were generated to assess the link between predicted and actual outcomes of patients. Decision curve analysis (DCA) was conducted to measure clinical utility and benefits by quantifying the net benefits at different threshold probabilities. The statistical significance levels were two-sided; a *P* < 0.05 was considered significant.

## 3 Results

### 3.1 Patient clinicopathological characteristics

**Figure 1:**
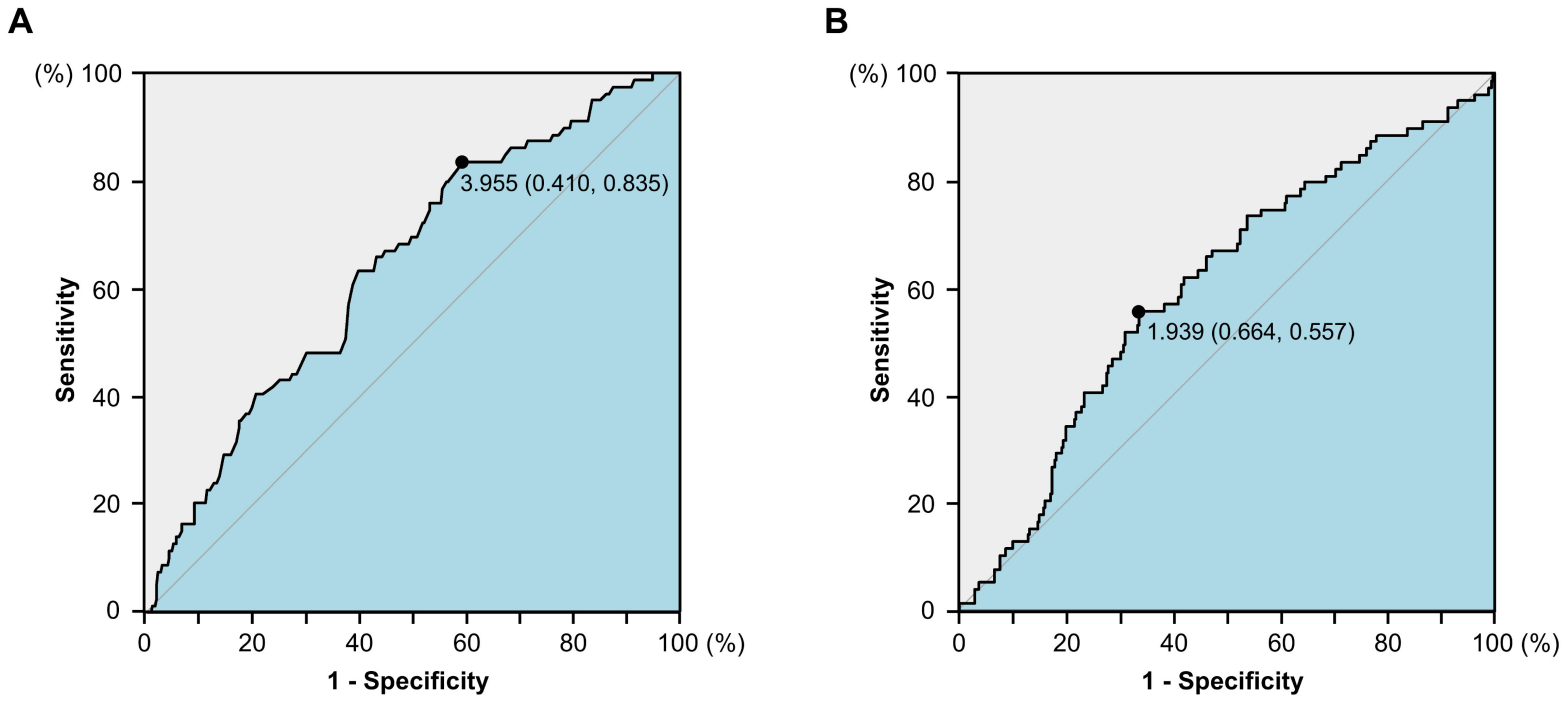
The receiver operating characteristics (ROC) curves for serum albumin level **(A)** and derived neutrophil-to-lymphocyte ratio **(B)** in the development cohort.

Baseline characteristics were summarized in **Table 1**. 457 of whom [190 (41.5%) women and 267 (58.5%) men] were enrolled in the development cohort. Their median age was 63 (range: 28-90). 87 patients had a history of smoking (current smoker or quit smoking but less than 2 years). 244 (53.3%) patients developed cancer in the rectum. 17.5% (80/457), 45.1% (206/457), and 37.4% (171/457) of the patients had stage I, II and III CRC. 263 (57.5%) of patients only underwent radical surgery. When a baseline albumin level of 3.955 g/dL and dNLR of 1.740 was used as the cut-off value (**Figure 1**), Alb-dNLR scores were calculated as described above. Subsequently, patients in the development cohort were split into two groups (0 vs. 1 or 2), and a baseline analysis was conducted. Eventually, 114 patients in the low Alb-dNLR score group and 343 in the high Alb-dNLR score group were analyzed. Compared to patients with low Alb-dNLR scores, patients with high Alb-dNLR scores were older (≥ 70 years old: 12.3% vs. 32.8%, *P* < 0.001).

**Table 1:** Clinicopathological characteristics of patients in development cohort.

Parameters	Total (N = 457)	Low Alb-dNLR Score (N = 114)	High Alb-dNLR Score (N = 343)	*P*
Sex				0.313
Female	190	52 (45.6)	138 (40.2)	
Male	267	62 (54.4)	205 (59.8)	
Age (years)				< 0.001
< 70	331	100 (87.7)	231 (67.3)	
≥ 70	126	14 (12.3)	112 (32.7)	
Smoking status				0.364
Never/Quitted	370	89 (78.1)	281 (81.9)	
Current	87	25 (21.9)	62 (18.1)	
Treatment				0.340
Operation	266	62 (54.4)	204 (59.5)	
Op + CT/RCT	191	52 (45.6)	139 (40.5)	
Tumor site				0.279
Ascending colon	81	13 (11.4)	68 (19.8)	
Transverse colon	13	3 (2.6)	10 (2.9)	
Descending colon	30	7 (6.1)	23 (6.7)	
Sigmoid colon	89	27 (23.7)	62 (18.1)	
Rectum	244	64 (56.1)	180 (52.5)	
TNM stage				0.133
I	80	27 (23.7)	53 (15.5)	
II	206	48 (42.1)	158 (46.1)	
III	171	39 (34.2)	132 (38.5)	
T stage				0.046
1	24	10 (8.8)	14 (4.1)	
2	71	23 (20.2)	48 (14.0)	
3	97	18 (15.8)	79 (23.0)	
4	265	63 (55.3)	202 (58.9)	
N stage				0.457
0	286	75 (65.8)	211 (61.5)	
1	107	27 (23.7)	80 (23.3)	
2	64	12 (10.5)	52 (15.2)	
Differentiation				0.897
Well-differentiated	47	12 (10.5)	35 (10.2)	
Moderately-differentiated	369	93 (81.6)	276 (80.5)	
Poorly-differentiated	41	9 (7.9)	32 (9.3)	

Alb-dNLR score, albumin-derived neutrophil-to-lymphocyte ratio score; Op, operation; CT, chemotherapy; RCT, radiochemotherapy.

### 3.2 Prognostic data

The median follow-up duration was 71 months (range: 1–120 months). By the end of follow-up, 94 patients had died, with 79 of these deaths attributed to recurrence. The cumulative CSS rates at 1, 3, and 5 years were 94.7%, 90.0%, and 84.4%, respectively. The OS rates at 1, 3, and 5 years were 93.7%, 88.4%, and 82.5%, respectively.

**Figure 2:**
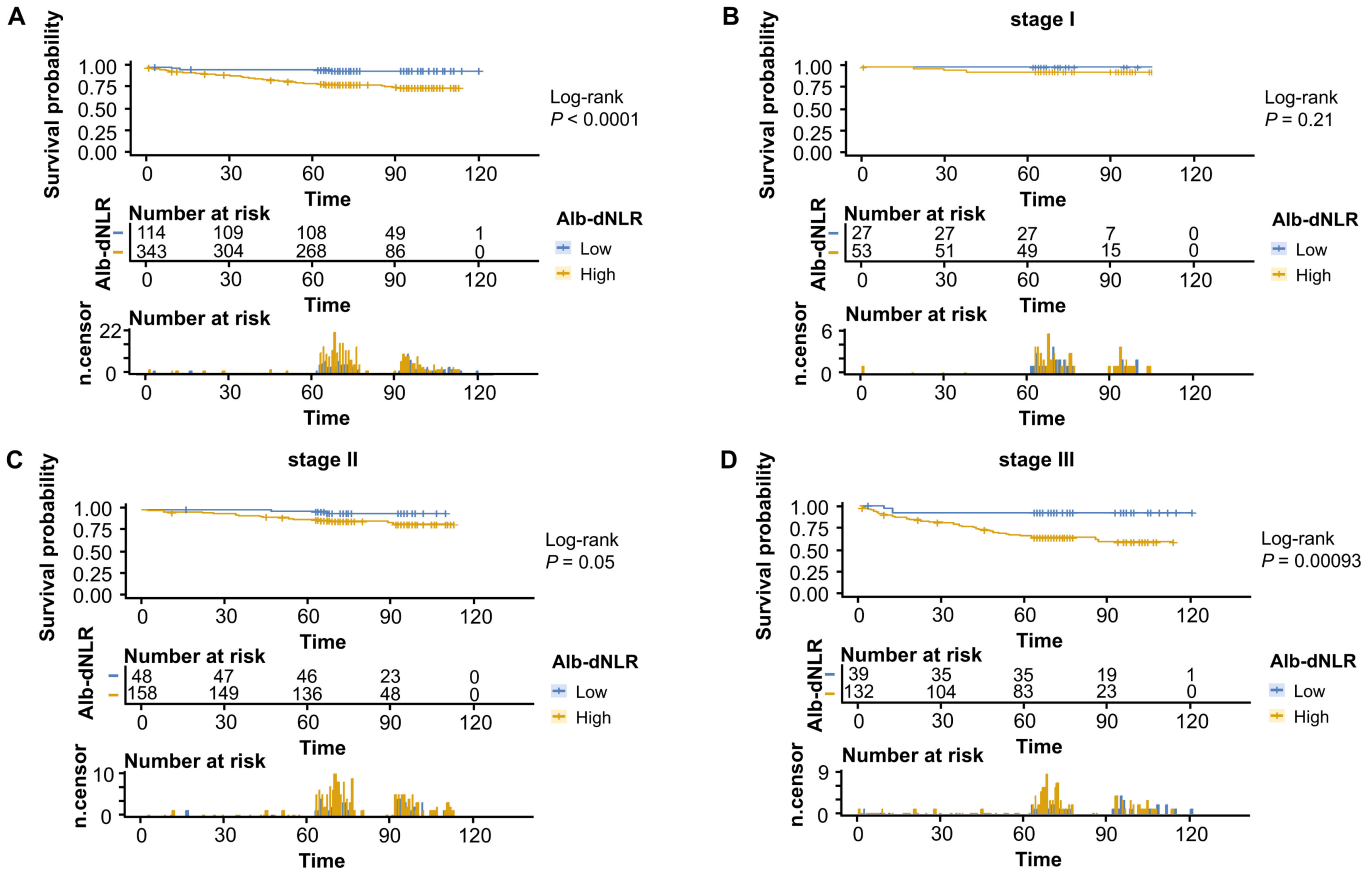
Kaplan-Meier survival curves for cancer-specific survival stratified by Alb-dNLR score groups in the development cohort. Median CSS was not reached in most subgroups except Stage III high Alb-dNLR score group (86 months, 95% CI: 86 months to not reached). Five-year CSS rates for low vs high Alb-dNLR score groups were: **(A)** All stages: 96.5% (95% CI: 93.1-99.9%) vs 80.5% (76.3-84.8%), P <.0001; **(B)** Stage I: 100% vs 94.2% (88.1-100%), P = 0.21; **(C)** Stage II: 97.9% (93.8-100%) vs 87.9% (82.9-93.1%), P = 0.05; **(D)** Stage III: 92.1% (83.9-100%) vs 65.8% (58.1-74.6%), P = 0.00093. Alb-dNLR, albumin-derived neutrophil-to-lymphocyte ratio score; CSS, cancer-specific survival; CI, confidence interval.

**Figure 3:**
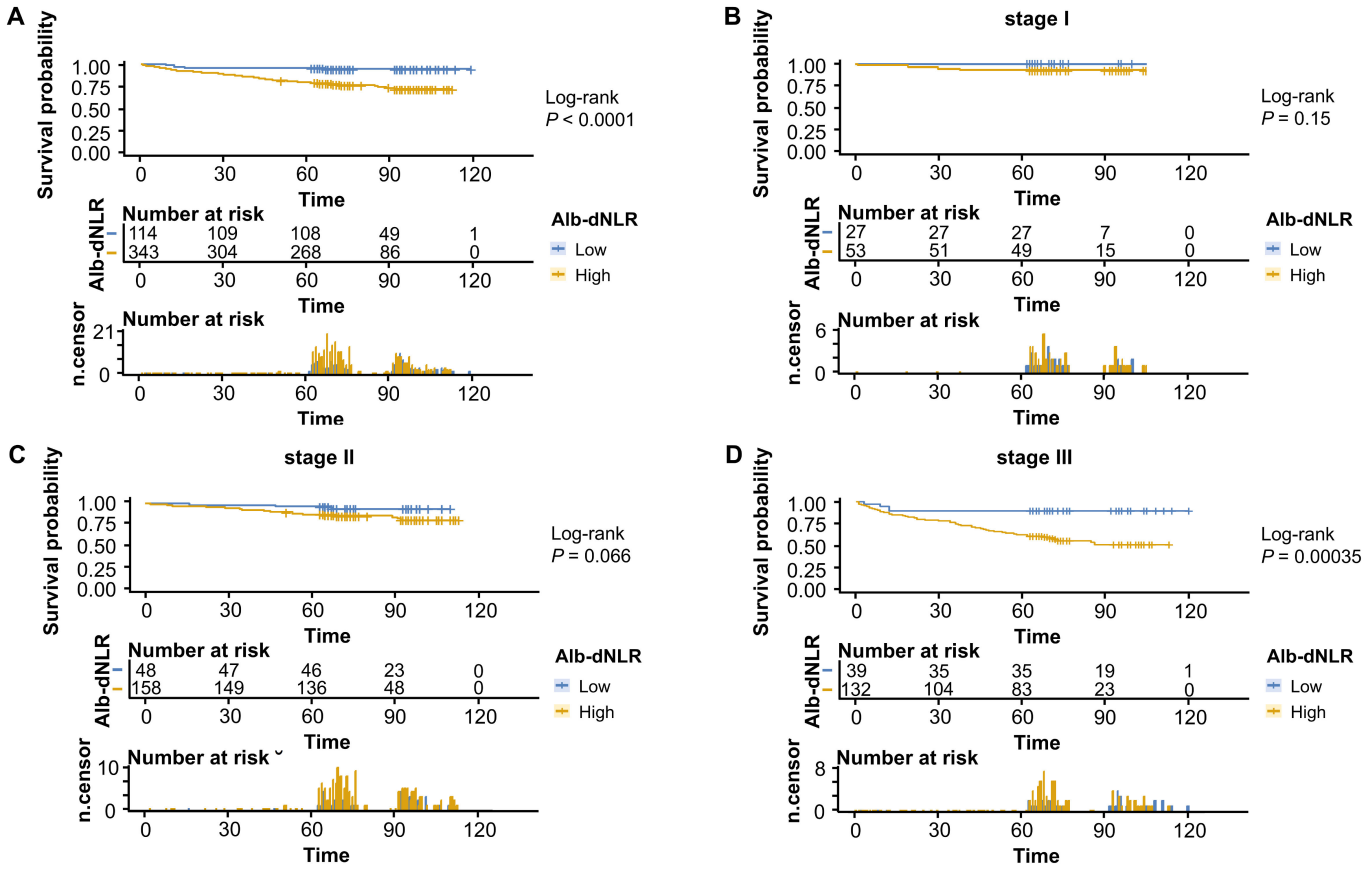
Kaplan-Meier survival curves for overall survival stratified by Alb-dNLR score groups in the development cohort. Median OS was not reached in most subgroups except Stage III high Alb-dNLR score group (72 months, 95% CI: 72 months to not reached). Five-year OS rates for low vs high Alb-dNLR score groups were: **(A)** All stages: 94.7% (95% CI: 90.7-98.9%) vs 78.4% (74.2-82.9%), P <.0001; **(B)** Stage I: 100% vs 92.5% (85.6-99.9%), P = 0.15; **(C)** Stage II: 95.8% (90.3-100%) vs 86.7% (81.5-92.2%), P = 0.066; **(D)** Stage III: 89.7% (80.7-99.8%) vs 62.9% (55.2-71.7%), P = 0.00035. Alb-dNLR, albumin-derived neutrophil-to-lymphocyte ratio score; OS, overall survival; CI, confidence interval.

The Kaplan-Meier survival curves and log-rank tests demonstrated a positive correlation between the Alb-dNLR score and the mortality risk. For all-stage patients, the 5-year CSS rate was significantly higher in the low Alb-dNLR score group compared to the high Alb-dNLR score group [96.5% (95% CI: 93.1-99.9%) vs 80.5% (76.3-84.8%), P < 0.0001], as was the 5-year OS rate [94.7% (90.7-98.9%) vs 78.4% (74.2-82.9%), P < 0.0001]. When stratified by stage, the Alb-dNLR score demonstrated significant discriminative ability for CSS in Stage II [97.9% (93.8-100%) vs 87.9% (82.9-93.1%), P = 0.05] and Stage III [92.1% (83.9-100%) vs 65.8% (58.1-74.6%), P = 0.00093] patients (**Figure 2**). Similarly, for OS, the score significantly stratified Stage III patients [89.7% (80.7-99.8%) vs 62.9% (55.2-71.7%), P = 0.00035], though differences in Stage I and II did not reach statistical significance (**Figure 3**).

### 3.3 Univariate and multivariate analysis

**Figure 4:**
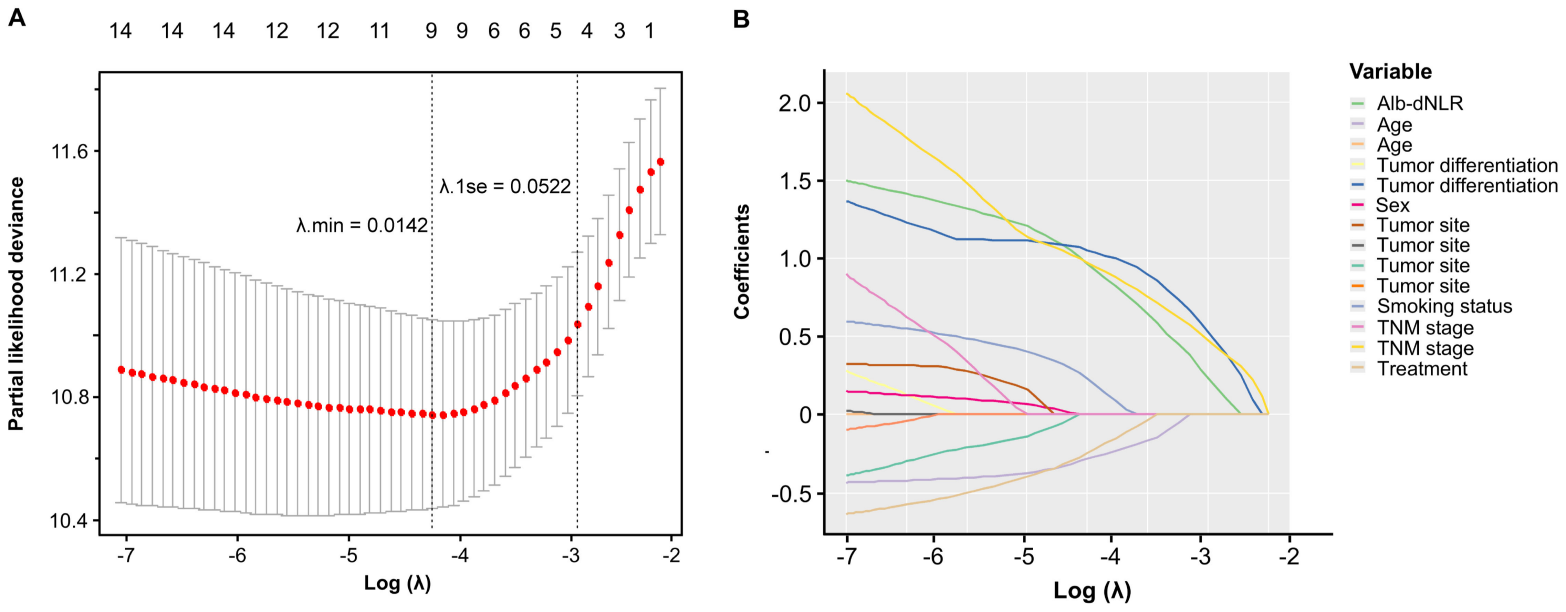
Selection of prognostic factors in the development cohort using LASSO regression. **(A)** the selection process of the optimal value of λ using a 10-time cross-validation method for tuning parameter selection in the LASSO model. **(B)** LASSO coefficient profiles.

The Cox proportional hazard model was employed to ascertain prognostic factors associated with CSS (**Table 2**) and OS (**Supplementary Table 1**). According to the univariate analysis, 6 variables were incorporated in multivariate Cox model, including Alb-dNLR score, age, treatment strategy, smoking status, pTNM stage, and tumor differentiation degree. In multivariate Cox regression analysis, Alb-dNLR score, pTNM stage, tumor differentiation degree, smoking status, and treatment strategy are mutual prognostic factors to CSS and OS. LASSO regression was utilized to filter potential risk factors for CSS, and a λ.1SE = 0.0522 was selected for the LASSO model when it provided decent performance but a minimum number of variables using a 10-fold cross-validation (**Figure 4A**), the coefficients profile of these variables versus log (λ) sequence was illustrated in **Figure 4B**. Eventually, the cross-validation identified 4 variables, including Alb-dNLR score, age, TNM stage, and tumor differentiation degree as independent factors for CSS, which was then utilized to construct a predictive nomogram.

**Table 2:** Univariate and multivariate analyses of parameters related with CSS in development cohort.

Parameters	Univariate analysis		Multivariate analysis	
	HR (95% CI)	P value	HR (95% CI)	P value
Sex				
Male vs. Female	1.331 (0.840-2.111)	0.224		
Age (years)				
≥ 70 vs.< 70	2.039 (1.304-3.189)	0.002	1.484 (0.898-2.451)	0.123
Alb-dNLR score				
High vs. Low	5.536 (2.237-13.700)	< 0.001	4.791 (1.904-12.056)	< 0.001
Smoking status				
Never/Quitted vs. Current	1.562 (0.940-2.595)	0.085	1.957 (1.141-3.356)	0.015
Treatment				
Op vs. Op + CT/RCT	0.630 (0.394-1.007)	0.053	1.905 (1.138-3.191)	0.014
Tumor site (colon)				
Ascending	Ref	0.795		
Transverse	1.086 (0.318-3.705)	0.896		
Descending	0.839 (0.310-2.274)	0.730		
Sigmoid	0.639 (0.305-1.338)	0.235		
Rectal	0.800 (0.456-1.406)	0.438		
TNM stage				
I	Ref	< 0.001	Ref	< 0.001
II	3.251 (0.981-10.767)	0.054	3.109 (0.909-10.631)	0.071
III	9.360 (2.921-29.994)	< 0.001	9.154 (2.760-30.357)	< 0.001
Differentiation				
Well	Ref	< 0.001	Ref	< 0.001
Moderately	1.809 (0.656-4.986)	0.252	1.302 (0.464-3.653)	0.617
Poorly	6.958 (2.354-20.569)	< 0.001	4.133 (1.369-12.474)	0.012

Alb-dNLR score, albumin-derived neutrophil-to-lymphocyte ratio score; Op, operation; CT, chemotherapy; RCT, radiochemotherapy; Ref, reference; HR, hazard ratio; CI, confidence interval.

### 3.4 Construction and validation of nomograms

**Figure 5:**
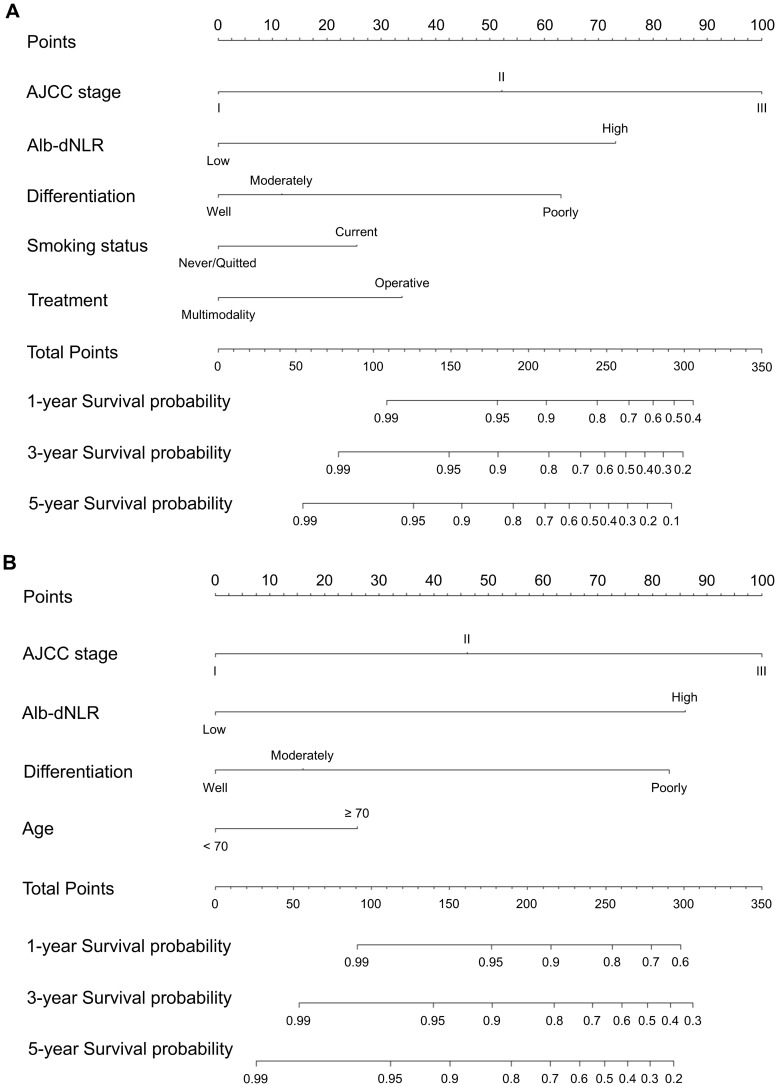
Constructed nomograms for prognostic prediction of 1,3,5-year cancer-specific survival of patients in development cohort. **(A)** Model A based on Cox proportional hazard ratio. **(B)** Model B based on LASSO regression.

Two novel nomograms (**Figures 5A, B**) were constructed according to prognostic factors for CSS screening by multivariable Cox (model A) and LASSO regression models (model B), respectively. Alb-dNLR score, TNM stage, tumor differentiation degree, smoking status and treatment strategy were incorporated in model A. TNM stage, age, Alb-dNLR score and differentiation were involved in model B. Each factor was assigned a score located on the variable axis; survival probability can be determined by calculating the sum of each variable’s score and plotting it on the total score axis.

**Figure 6:**
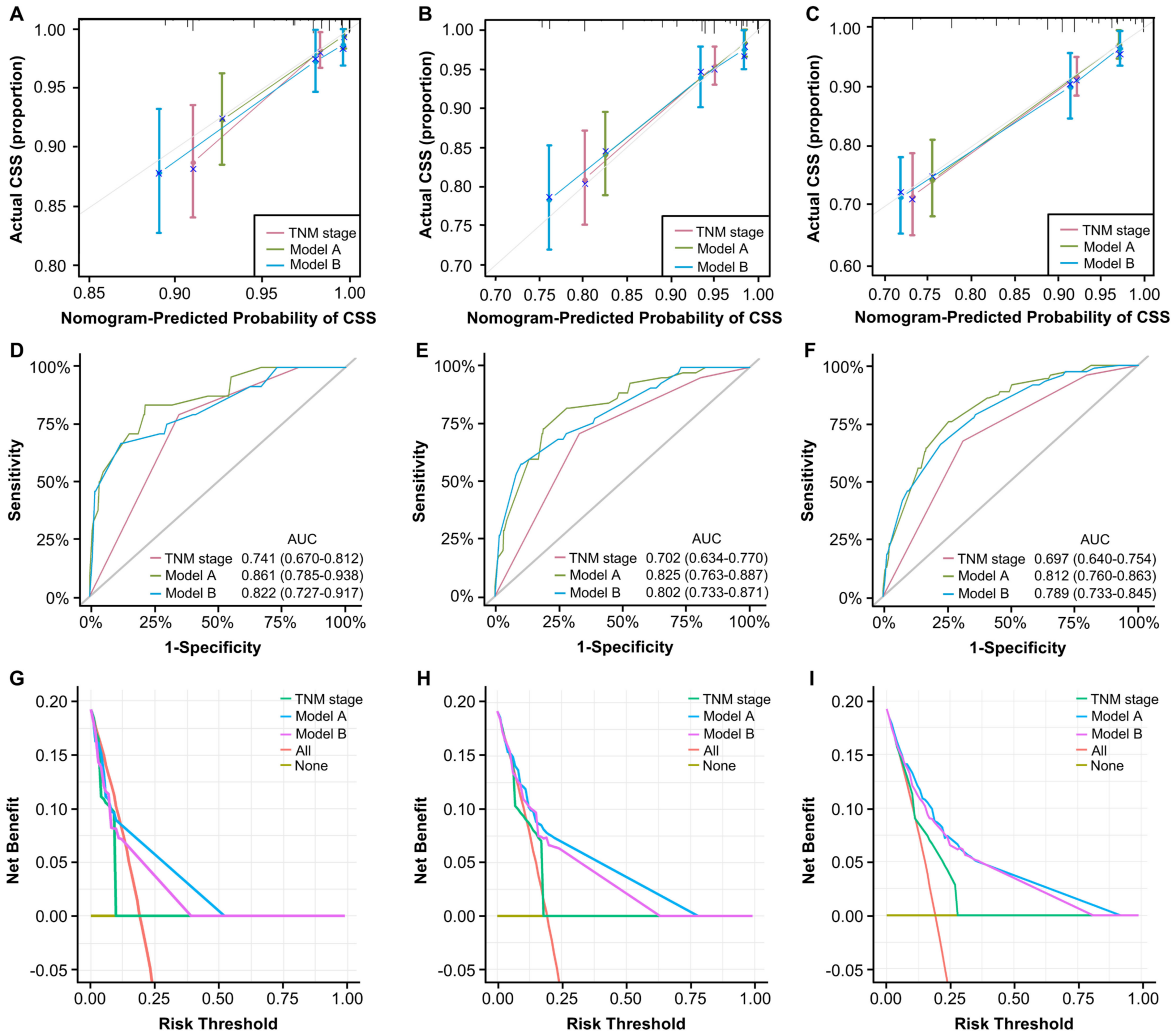
Validation of constructed nomograms for 1, 3, and 5-year CSS in development cohort. Calibration curves for 1 **(A)**, 3 **(B)**, and 5-year CSS prediction **(C)** based on non-metastatic CRC patients. Time-dependent ROC analyses of 1 **(D)**, 3 **(E)**, and 5-year CSS prediction **(F)** based on TNM stage, model A, and model B DCA curves for 1 **(G)**, 3 **(H)**, and 5-year CSS prediction **(I)** based on TNM stage, model A and model B.

The assessment and verification of a nomogram encompass three components: discriminative ability, calibration accuracy, and clinical utility. First, C-indexes demonstrated that our nomograms perform better than TNM staging system (0.785, 0.767 vs. 0.680). Then, calibration curves were generated and demonstrate satisfactory consistencies between the predictions and actual outcomes in 1, 3, and 5 years of CSS (**Figures 6A-C**). Based on time-ROC, our nomograms displayed superior performance to the TNM staging system, as the area under the time-dependent ROC curves (AUC) generated significantly surpass that of TNM staging, indicating favorable discriminative ability (**Figures 6D-F**).

The DCA curves evaluate models in terms of clinical outcome, demonstrating a consistent increase in clinical net benefits over time, reaffirming the enduring clinical significance of our nomograms for CSS and OS. Our models displayed better clinical net advantages that grew over time compared to the TNM staging system (**Figures 6G-I**). These results have confirmed the robust predictive capacity, accuracy, and enhanced clinical applicability of our models in comparison to the AJCC TNM stage system.

### 3.5 Further validation in an independent set of cases

**Figure 7:**
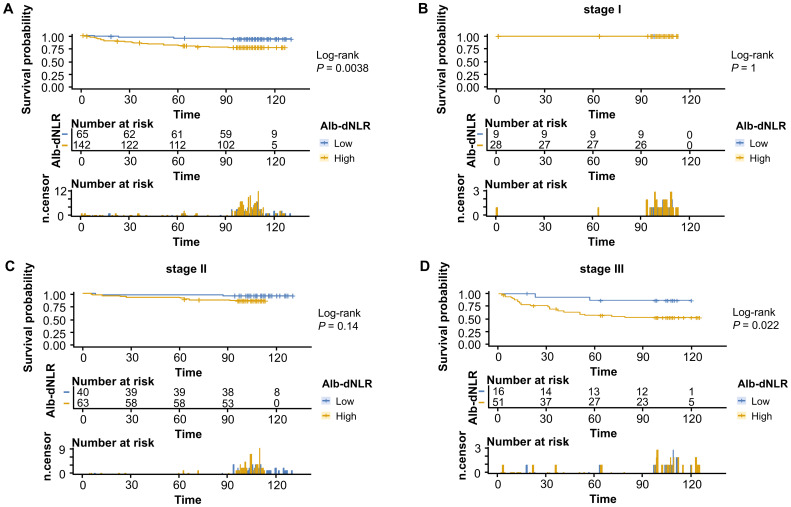
Kaplan-Meier survival curves for cancer-specific survival stratified by Alb-dNLR score groups in the validation cohort. Median CSS was not reached in any subgroup during the follow-up period. Five-year CSS rates for low vs high Alb-dNLR score groups were: **(A)** All stages: 95.3% (95% CI: 90.3-100%) vs 80.6% (74.3-87.5%), P = 0.0038; **(B)** Stage I: 100% vs 100%, P = 1.0; **(C)** Stage II: 97.5% (92.8-100%) vs 90.5% (83.5-98.0%), P = 0.14; **(D)** Stage III: 86.7% (71.1-100%) vs 57.2% (44.8-72.9%), P = 0.022. Alb-dNLR, albumin-derived neutrophil-to-lymphocyte ratio score; CSS, cancer-specific survival; CI, confidence interval.

**Figure 8:**
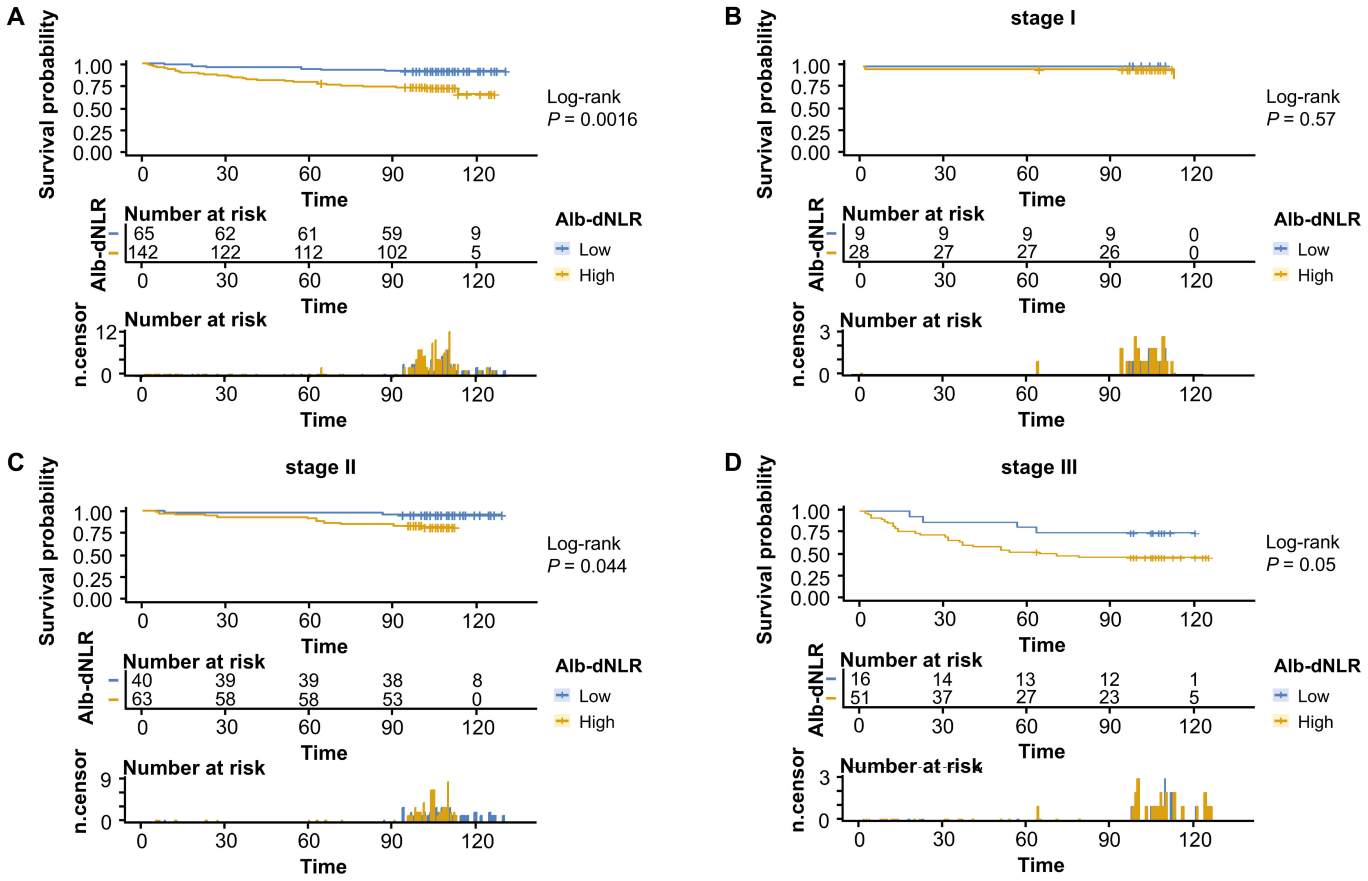
Kaplan-Meier survival curves for overall survival stratified by Alb-dNLR score groups in the validation cohort. Median OS was not reached in most subgroups except Stage I high Alb-dNLR score group (113 months, 95% CI: 113 months to not reached) and Stage III high Alb-dNLR score group (71 months, 95% CI: 37 months to not reached). Five-year OS rates for low vs high Alb-dNLR score groups were: **(A)** All stages: 93.9% (95% CI: 88.2-99.9%) vs 78.2% (71.7-85.3%), P = 0.0016; **(B)** Stage I: 100% vs 96.4% (89.8-100%), P = 0.57; **(C)** Stage II: 97.5% (92.8-100%) vs 90.5% (83.5-98.0%), P = 0.044; **(D)** Stage III: 81.3% (64.2-100%) vs 52.9% (40.9-68.6%), P = 0.05. Alb-dNLR, albumin-derived neutrophil-to-lymphocyte ratio score; OS, overall survival; CI, confidence interval.

To comprehensively assess and validate the prognostic value of Alb-dNLR score and the predictive, discriminatory, and clinical advantages of developed nomograms, we included 207 patients who had undergone radical surgery for CRC based on the previously outlined criteria, a validation cohort was established and dichotomized as shown in **Supplementary Table 2**. Kaplan-Meier curves revealed that, in line with the results in the development cohort, elevated Alb-dNLR score remained significantly associated with inferior CSS (*P* = 0.0038) (**Figure 7**) and OS (*P* = 0.0016) in the validation cohort (**Figure 8**), particularly among those with stage III disease (*P* = 0.022, *P* = 0.05). This trend is also exhibited in the OS of stage II CRC patients (*P* = 0.044), albeit not statistically significant in CSS.

**Figure 9:**
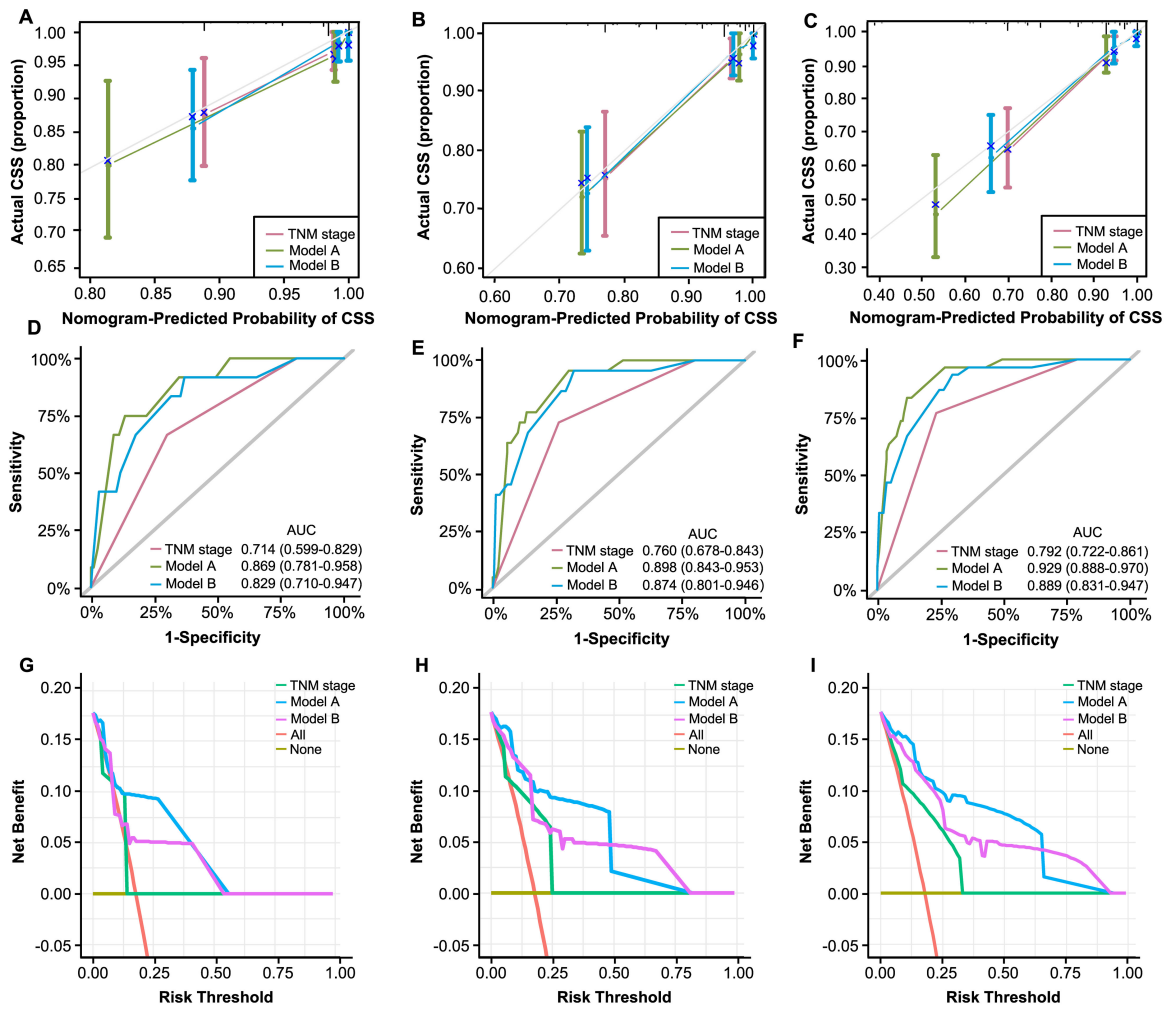
Validation of constructed nomograms for 1, 3, and 5-year CSS in the validation cohort. Calibration curves for 1 **(A)**, 3 **(B)**, and 5-year CSS prediction **(C)** based on non-metastatic CRC patients. Time-dependent ROC analyses of 1 **(D)**, 3 **(E)**, and 5-year CSS prediction **(F)** based on TNM stage, model A, and model B DCA curves for 1 **(G)**, 3 **(H)**, and 5-year CSS prediction **(I)** based on TNM stage, model A and model B.

We applied the novelly built nomograms in the validation cohort and conducted a set of analyses to comprehensively explore their predictive performance, discrimination, consistency, and clinical utility. First, the C-indexes of our models, 0.891 and 0.852, significantly surpassed 0.743 of TNM stage, demonstrating the superior performance of our nomograms. Based on 1, 3, and 5 years of CSS, a series of calibration curves were generated (**Figures 9A-C**), indicating a sound consistency. In the time-ROC analysis (**Figures 9D-F**), the AUCs of our models were significantly greater than those of TNM stage, indicating their outstanding discriminative abilities. Furthermore, the DCA curves showed that models A and B produced superior clinical net benefits for CSS in comparison to TNM stage (**Figures 9G-I**).

## 4 Discussion

Accumulating evidence suggesting inflammatory and nutrition-based markers as reliable prognostic factors in cancer patients (22, 23). Over the past decade, biomarkers like C-reactive protein (CRP) (24, 25), GPS (26), mGPS (27), and CALLY index (13), have been extensively investigated. However, these indicators require additional CRP blood tests beyond routine bloodwork, limiting their accessibility. PNI combines albumin and lymphocyte count but is weighted predominantly toward albumin (coefficient 10 versus 0.005), limiting its sensitivity in capturing dynamic inflammatory responses (28, 29). The Systemic Immune-Inflammation Index (SII), calculated from platelet, neutrophil, and lymphocyte counts, reflects platelet-mediated inflammatory pathways and has been extensively applied in colorectal cancer prognosis (30), yet lacks nutritional parameters such as albumin.

In contrast, Alb-dNLR score integrates nutritional and immune-inflammatory status, simultaneously addressing two critical aspects of tumor progression. More importantly, all components are derived from routine blood tests without additional laboratory requirements, offering superior accessibility and cost-effectiveness. The dNLR, initially proposed by Proctor et al. (10), can be calculated using only total leukocyte and neutrophil counts and has demonstrated prognostic value in ovarian cancer (31) and pancreatic cancer (32). It exhibits comparable prognostic value to NLR (10, 33) but does not require a lymphocyte count, enhancing its accessibility and ease of calculation. Neutrophils facilitate tumor development through various mechanisms, including promoting tumor angiogenesis, inducing immune suppression, and secreting cytokines (34). Conversely, serum albumin reflects nutritional status, liver function, and systemic inflammation, making it a valuable prognostic marker (35).Hypoalbuminemia may result from malnutrition due to gastrointestinal symptoms or from tumor-derived cytokines such as interleukin-6 (36). Beyond its role as a nutritional marker, albumin participates in binding and transporting various ligands (37, 38), and exerts antioxidant (39), anti-inflammatory (35) and anticoagulant effects (40). Consequently, decreased albumin levels may impair drug metabolism, increase oxidative stress, and heighten susceptibility to thrombosis and inflammation.

The Alb-dNLR score was first established by Chen et al. (15) to measure disease activity in rheumatoid arthritis (RA) patients, suggesting its potential for revealing chronic inflammatory status. Studies by Nakanishi et al. (41) found that an elevated Alb-dNLR score was an independent prognostic factor for recurrence-free survival (RFS) in patients with locally advanced rectal cancer (LARC) treated with neoadjuvant chemoradiotherapy. Notably, age (P = 0.214) and tumor differentiation degree (P = 0.532) were not found to be correlated with patients’ survival in their study. In addition, the clinical performance of their research would be limited due to its sample size (n = 69) and lack of patients’ life history (e.g., smoking history). In contrast, our study retrospectively recruited a total of 664 CRC patients across the entire spectrum of non-metastatic CRC (stages I-III), meanwhile, two sets of multivariate analyses utilizing Cox and LASSO regression models were conducted. They confirmed Alb-dNLR score as an independent risk factor for CSS (HR = 4.791, P < 0.001) and OS (HR = 3.952, P = 0.001) in CRC patients.

Interestingly, while Cox regression identified smoking status and treatment strategy as independent prognostic factors, these variables were excluded from the LASSO model. This discrepancy may derive from their different analytical objectives: Cox regression prioritizes identifying independent risk factors through hypothesis testing, whereas LASSO emphasizes predictive accuracy and model parsimony through regularization (42, 43). The LASSO model achieved a C-index of 0.767, only marginally lower than the full Cox model (0.785), indicating comparable discriminative performance. More importantly, the LASSO model achieved this performance with fewer variables, enhancing clinical practicality by reducing data collection burden while maintaining comparable predictive performance.

Recently, nomograms have been widely used as visual tools for individualized prediction of the survival probability of patients by integrating multidimensional factors (44). Nomograms can incorporate TNM staging information and multidimensional factors such as age, immune status, and inflammatory indicators, thereby more precisely capturing the complexity of the disease and offering personalized prognostic predictions. A retrospective study included 400 eligible patients (45) and built nomograms that combine nutritional (body mass index, albumin level) and inflammatory markers (NLR, neutrophil counts). However, this study lacked sufficient sample size for further validation, and the discrimination of models was not significantly better than TNM staging according to the C-indexes (0.820 vs. 0.789 for OS; 0.803 vs. 0.784 for disease-free survival (DFS). In contrast, we recruited a development cohort of 457 patients, developed two nomograms using Cox and LASSO regression models. More importantly, an independent validation cohort of 207 patients spanning all stages of non-metastatic CRC (stage I-III) was recruited to reduce statistical screening bias and rigorously validate the predictive models across the full spectrum of resectable disease.

Beyond their statistical performance, our Alb-dNLR score and derived nomograms provide practical tools for postoperative risk stratification in patients with non-metastatic CRC. Current guidelines recommend uniform surveillance based primarily on TNM stage (46), however, our findings demonstrate that Alb-dNLR score enables further risk stratification within TNM stages, with 5-year CSS rates differing by approximately 16% in all-stage patients and > 26% in Stage III patients. For low-risk patients, particularly those with Stage I-II disease showing 5-year survival exceeding 95%, de-escalated surveillance may reduce healthcare burden without compromising outcomes (47). Conversely, high-risk patients, especially those with Stage III disease, may benefit from intensified monitoring or treatment optimization. Importantly, the Alb-dNLR score can be calculated from routine blood tests without additional cost, facilitating its implementation.

An important translational question is whether Alb-dNLR score represents a modifiable therapeutic target beyond its prognostic value. Both components are potentially responsive to nutritional and anti-inflammatory interventions. While preliminary evidence from gastrointestinal cancer patients suggests that preoperative immune-nutrition and agents such as aspirin and dexamethasone can improve inflammatory parameters (48–51), prospective trials are needed to establish whether such optimization translates to improved survival outcomes in CRC.

While our nomograms demonstrated superior discriminative ability, they were developed primarily using clinicopathological and routine laboratory parameters without molecular biomarkers, such as microsatellite instability (MSI) (52). MSI-high (MSI-H) tumors, accounting for approximately 15-17% of CRCs, are associated with favorable prognosis, particularly in stage II-III disease, and exhibit distinct responses to chemotherapy and immunotherapy (53, 54), and we acknowledge that the inclusion of MSI status and other molecular markers could potentially enhance model performance and refine risk stratification. The absence of molecular markers in our study reflects the temporal evolution of clinical practice: routine MSI testing became standard in China only in 2023 (55), after the beginning of follow-up period. However, established tools including AJCC staging and earlier nomograms (56, 57), were similarly developed without molecular markers yet remain clinically useful, demonstrating that robust prognostic information can be derived from comprehensive clinicopathological data. Future models should integrate both conventional and molecular features to optimize personalized risk prediction.

However, limitations still occur. First, this single-center retrospective study may introduce selection bias. External validation in multi-center cohorts is essential to confirm generalizability. Second, absence of molecular biomarkers such as MSI and RAS/BRAF mutations represents a limitation, though our model incorporates tumor location (58) and differentiation (59) that may partially correlate with molecular subtypes. Third, hematological parameters were assessed only at baseline; dynamic changes in Alb-dNLR score may offer additional prognostic insights.

## 5 Conclusion

To summarize, Alb-dNLR score could be a convenient and effective prognostic marker for CSS and OS for non-metastatic CRC patients. Furthermore, our nomograms incorporating Alb-dNLR score and other clinicopathological risk factors were highly efficacious in predicting survival outcomes in patients with non-metastatic CRC.

## Data Availability

The raw data supporting the conclusions of this article will be made available by the authors, without undue reservation.
