# Supplementary material for: Validated nomograms for non-metastatic colorectal cancer prognosis prediction: a population-based study
Source: Front Oncol. 2025 Oct 24;15:1691693. doi: 10.3389/fonc.2025.1691693 (PMC12591884; doi:10.3389/fonc.2025.1691693)
Supplement: Supplementary file 1 [file Table1.docx]

|  | **Univariate analysis** | | **Multivariate analysis** | |
| --- | --- | --- | --- | --- |
|  | **HR (95% CI)** | **P value** | **HR (95% CI)** | **P value** |
| **Sex** |  |  |  |  |
| Male vs. Female | 1.234 (0.812-1.874) | 0.325 |  |  |
| **Age (years)** |  |  |  |  |
| ≥ 70 vs. < 70 | 2..013 (1.336-3.036) | < 0.001 | 1.400 (0.887-2.209) | 0.148 |
| **Alb-dNLR score** |  |  |  |  |
| High vs. Low | 4.690 (2.170-10.134) | < 0.001 | 3.953 (1.799-8.689) | < 0.001 |
| **Smoking status** |  |  |  |  |
| Never/Quitted vs. Current | 1.583 (0.995-2.518) | 0.052 | 2.072 (1.269-3.384) | 0.004 |
| **Treatment** |  |  |  |  |
| Op. vs. Op.+ CT/RCT | 1.940 (1.241-3.033) | 0.004 | 0.412 (0.254-0.667) | < 0.001 |
| **Tumor site (colon)** |  |  |  |  |
| Ascending | Ref | 0.914 |  |  |
| Transverse | 1.376 (0.466-4.067) | 0.563 |  |  |
| Descending | 0.952 (0.378-2.398) | 0.917 |  |  |
| Sigmoid | 0.856 (0.441-1.660) | 0.645 |  |  |
| Rectal | 0.879 (0.512-1.509) | 0.640 |  |  |
| **TNM stage** |  |  |  |  |
| I | Ref | < 0.001 | Ref | < 0.001 |
| II | 2.803 (0.985 -7.973) | 0.053 | 3.124 (1.063 -9.184) | 0.038 |
| III | 8.361 (3.040-22.986) | < 0.001 | 9.623 (3.384-27.365) | < 0.001 |
| **Differentiation** |  |  |  |  |
| Well | Ref | < 0.001 | Ref | < 0.001 |
| Moderately | 1.452 (0.630-3.344) | 0.381 | 1.010 (0.430-2.374) | 0.981 |
| Poorly | 4.786 (1.910-11.992) | < 0.001 | 2.747 (1.073-7.032) | 0.035 |

**Supplemental Table 1 Univariate and multivariate analyses of parameters related with OS in development cohort**

Abbreviations: Alb-dNLR, albumin-derived neutrophil-to-lymphocyte ratio score; Op, operation; CT, chemotherapy; RCT, radiochemotherapy; HR, hazard ratio; CI, confidence interval.
