# Supplementary material for: Validated nomograms for non-metastatic colorectal cancer prognosis prediction: a population-based study
Source: Front Oncol. 2025 Oct 24;15:1691693. doi: 10.3389/fonc.2025.1691693 (PMC12591884; doi:10.3389/fonc.2025.1691693)
Supplement: Supplementary file 2 [file Table2.docx]

**Supplemental Table 2 Clinicopathological characteristics of patients in validation cohort**

| **Parameters** | **Total**  **N = 207** | **Low Alb-dNLR**  **N = 65** | **High Alb-dNLR**  **N = 142** | ***P*** |
| --- | --- | --- | --- | --- |
| **Sex** |  |  |  | 0.064 |
| Female | 83 | 20 (30.8) | 63 (44.4) |  |
| Male | 124 | 45 (69.2) | 79 (55.6) |  |
| **Age (years)** |  |  |  | 0.078 |
| < 70 | 163 | 56 (86.2) | 107 (75.4) |  |
| ≥ 70 | 44 | 9 (13.8) | 35 (24.6) |  |
| **Smoking status** |  |  |  | 0.019 |
| Never/Quitted | 169 | 47 (72.3) | 122 (85.9) |  |
| Current | 38 | 18 (27.7) | 20 (14.1) |  |
| **Treatment** |  |  |  | 0.086 |
| Operation | 123 | 33 (50.8) | 90 (63.4) |  |
| Op + CT/RCT | 84 | 32 (49.2) | 52 (36.6) |  |
| **Tumor site** |  |  |  | 0.299 |
| Ascending colon | 38 | 7 (10.8) | 31 (21.8) |  |
| Transverse colon | 10 | 4 (6.2) | 6 (4.2) |  |
| Descending colon | 10 | 2 (3.1) | 8 (5.6) |  |
| Sigmoid colon | 28 | 9 (13.8) | 19 (13.4) |  |
| Rectum | 121 | 43 (66.2) | 78 (54.9) |  |
| **TNM stage** |  |  |  | 0.072 |
| I | 37 | 9 (13.8) | 28 (19.7) |  |
| II | 103 | 40 (61.5) | 63 (44.4) |  |
| III | 67 | 16 (24.6) | 51 (35.9) |  |
| **T stage** |  |  |  | 0.237 |
| 1 | 17 | 5 (7.7) | 12 (8.5) |  |
| 2 | 24 | 5 (7.7) | 19 (13.4) |  |
| 3 | 36 | 16 (24.6) | 20 (14.1) |  |
| 4 | 130 | 36 (60.0) | 91 (64.1) |  |
| **N stage** |  |  |  | 0.262 |
| 0 | 140 | 49 (75.4) | 91 (64.1) |  |
| 1 | 44 | 11 (16.9) | 33 (23.2) |  |
| 2 | 23 | 5 (7.7) | 18 (12.7) |  |
| **Differentiation** |  |  |  | 0.483 |
| Well differentiated | 24 | 5 (7.7) | 19 (13.4) |  |
| Moderately differentiated | 160 | 52 (80.0) | 108 (76.1) |  |
| Poorly differentiated | 23 | 8 (12.3) | 15 (10.6) |  |

Abbreviations: Alb-dNLR, albumin-derived neutrophil-to-lymphocyte ratio score; Op, operation; CT, chemotherapy; RCT, radiochemotherapy; HR, hazard ratio; CI, confidence interval.
